# Dynamic Hand Gesture Recognition Based on a Leap Motion Controller and Two-Layer Bidirectional Recurrent Neural Network

**DOI:** 10.3390/s20072106

**Published:** 2020-04-08

**Authors:** Linchu Yang, Ji’an Chen, Weihang Zhu

**Affiliations:** 1 Department of Mechanical Engineering; Jiangsu University of Science and Technology, Zhenjiang 212003, China; yanglinchu@just.edu.cn; 2 Department of Engineering Technology; University of Houston, Houston, TX 77204, USA; wzhu21@central.uh.edu

**Keywords:** hand gesture recognition, leap motion controller (LMC), recurrent neural network (RNN)

## Abstract

Dynamic hand gesture recognition is one of the most significant tools for human–computer interaction. In order to improve the accuracy of the dynamic hand gesture recognition, in this paper, a two-layer Bidirectional Recurrent Neural Network for the recognition of dynamic hand gestures from a Leap Motion Controller (LMC) is proposed. In addition, based on LMC, an efficient way to capture the dynamic hand gestures is identified. Dynamic hand gestures are represented by sets of feature vectors from the LMC. The proposed system has been tested on the American Sign Language (ASL) datasets with 360 samples and 480 samples, and the Handicraft-Gesture dataset, respectively. On the ASL dataset with 360 samples, the system achieves accuracies of 100% and 96.3% on the training and testing sets. On the ASL dataset with 480 samples, the system achieves accuracies of 100% and 95.2%. On the Handicraft-Gesture dataset, the system achieves accuracies of 100% and 96.7%. In addition, 5-fold, 10-fold, and Leave-One-Out cross-validation are performed on these datasets. The accuracies are 93.33%, 94.1%, and 98.33% (360 samples), 93.75%, 93.5%, and 98.13% (480 samples), and 88.66%, 90%, and 92% on ASL and Handicraft-Gesture datasets, respectively. The developed system demonstrates similar or better performance compared to other approaches in the literature.

## 1. Introduction

The main purpose of human–computer interaction is to allow users to freely control the device with some simple operations [1]. The human–computer interaction techniques include face recognition, language recognition, text recognition, and so on. As one of the important and powerful interaction methods, dynamic hand gesture recognition has attracted wide attention and been used in various fields, such as the video game industry, food industry, and machinery industry [2,3,4].

Dynamic gesture recognition can be used in many applications, such as virtual reality [5,6], the remote operation of robot [7,8], video games [9,10], sign language interpretation [11,12], and so on. In general, dynamic hand gesture recognition can be mainly divided into vision-based gesture recognition and wearable device-based gesture recognition. In the past, many researches on dynamic gesture recognition basically used a monocular camera [13] to capture images of dynamic gestures, segmented images, and extracted the gesture model from a series of frames. One disadvantage of this method is that a large amount of computation is required to segment the hand information. The other method adopted a data glove [14] to collect data that focuses on the coordinates of palms and fingertips and the bending degrees of finger joints. It can intuitively obtain hand information without a lot of calculation. However, a user must wear gloves, and people using the same device may get certain health issues. The recent years have seen some novel sensors, such as Leap Motion Controllers (LMC) [15] and Microsoft Kinect [16]. These sensors do not need to be worn and a user only need to put the hand in the appropriate acquisition space above the sensor to allow the three-dimensional information of the hand to be captured in its three-dimensional coordinate system. This is a significant contribution to the dynamic gesture recognition.

Currently, the majority of studies on the dynamic gesture recognition are based on the Support Vector Machine, Hidden Markov Model, Common Neural Network, Rule-based Modeling techniques and so on.

In the previous works, Support Vector Machine, Dynamic Time Warping, and Hidden Markov Model were commonly adopted. For example, Xu et al. displayed a method of feature extraction for dynamic hand gesture recognition based on the LMC, which ensured that the lengths of different dynamic hand gesture samples could be the same [17]. Next, a classifier based on the Support Vector Machine was used to recognize dynamic gestures. At last, the recognition accuracies for Arabic numbers (0-9), based on two different ranging criterions, were 90.75% and 91.375%, respectively. Karthick et al. presented a system which can transform Indian Sign Language to text [18]. In addition, the hand gestures were also collected by the LMC. Afterwards, they translated dynamic hand gestures into text by combining an Intelligent Sensing algorithm with Dynamic Time Warping. Moreover, Schramm obtained many patterns by tracking hand position through an RGB-D camera [19]. After that, researchers used a probabilistic model based on Dynamic Time Warping to recognize and evaluate patterns. The LMC had also been used in [20] and a method was proposed for segmenting and recognizing texts that are drawn by fingers in the 3D coordinate system. By using a heuristic analysis of stroke length between two consecutive words, the successive text was segmented into words. Then, the Hidden Markov Model was used to identify each segmented word. As a result, an accuracy of 81.25% was obtained on word recognition.

In some other works, different technologies were extended. Lu et al. proposed a special feature vector which represents the gesture and presented a Hidden Conditional Neural Field that incorporates the gate function of neural networks to recognize dynamic hand gestures with an LMC [21]. With hand information, the vector of features was collected and input into the proposed model to recognize dynamic gestures. Finally, the method was tested with two dynamic hand gesture datasets, and the accuracy was 89.5% on a subset of American Sign Language and 95% on the Handicraft-Gesture dataset. Zhang et al. presented a gesture recognition system that combines the modeling ability of the Hidden Markov Model on time-based sequences with the interpretability of the Classification and Regression Tree to achieve fast classification and regression [22]. Features were divided into four types, namely finger shape, palm ball radius, palm normal vector, and palm displacement vector. In the first part, a Hidden Markov Model was built for each type. Next, the output of the first part was regarded as the input of the Classification and Regression Tree model in the second part. The output of the second part was the recognition result. Finally, the experiments showed that the accuracy was higher than that of traditional single channel Hidden Markov Model.

In recent years, more and more researchers have used the LMC and some advanced algorithms to recognize dynamic gestures. Avola et al. presented a deep Recurrent Neural Network model to identify dynamic hand gestures from American Sign Language [23]. The proposed method was tested by a challenging dataset which consists of 12 dynamic hand gestures from the American Sign Language and the final accuracy was 96%. Zeng et al. developed a new approach to recognize dynamic hand gestures for Arabic numbers (0–9) and capital English alphabets (A–Z) [24]. In the first step, dynamic hand gesture features were derived from the LMC. Secondly, the authors used the radial basis function neural network to approximate the hand motion dynamics underlying the motion patterns of different hand gestures. The difference between motion dynamics was used to distinguish between different hand gestures. Finally, by using the leave-one-person-out, 2-fold, and 10-fold cross-validation, the recognition accuracy results for 0–9 are 94.2%, 95.1%, and 90.2%, respectively, and for A–Z are 89.2%, 92.9%, and 86.4%, respectively. Mittal et al. proposed a recognition system for continuous sign language by using LMC [25]. In the system, the authors presented a modified Long Short-Term Memory model which has three input gates and an output gate, adding a 2D-Convolutional Neural Network to the input gate that receives feature inputs. At last, the system had been tested with Indian Sign Language and got the average accuracies of 89.5% and 72.3% on isolated sign words and signed sentences, respectively. The Arabic Sign Language recognition system with two LMCs was shown in [26]. Deriche et al. adopted the Linear Discriminant Analysis approach and a Bayesian approach that contains the Gaussian Mixture Model. As a result, an accuracy of nearly 92% was obtained.

In order to improve the performance of the dynamic hand gesture recognition on American Sign Language (ASL) and Handicraft Gesture datasets from several works, we present a gesture recognition system in this paper, which consists of the LMC and the two-layer Bidirectional Recurrent Neural Network (BRNN). In the first stage, the proposed algorithm accurately determines the start and end of dynamic hand gestures by calculating the changes in hand rotation angle and palm speed between two adjacent frames, which can ensure the validity of features. Then, to obtain a better model, the features of a single finger and adjacent fingers are introduced into the input vector of the model. In the next stage, we compare the effects of changes in the hyper-parameter on the accuracy of the classifier and improve the performance of the model. In the third stage, the validation and comparison are performed on the proposed system. Here, the challenges are how to collect real-time dynamic hand gestures efficiently and how to search for a better model with hyper-parameters. The first problem is solved, to a large extent, by using an algorithm to discriminate hand movement, and then the second problem is tackled by using a two-layer BRNN. In summary, the contributions of the proposed system are shown:

Based on the LMC, we propose a method to accurately determine when to start and end collecting dynamic gestures through its C++ function library.

The selection of features, based on fingers and palm, are highly discriminative for recognizing some gestures from the ASL and Handicraft-Gesture datasets.

For the first time, we combine a two-layer BRNN with the special features extracted by the LMC in the field of the dynamic gesture recognition. Moreover, the accuracy is higher than that reported in similar works found in the literature.

## 2. Materials and Methods

**Figure 1:**
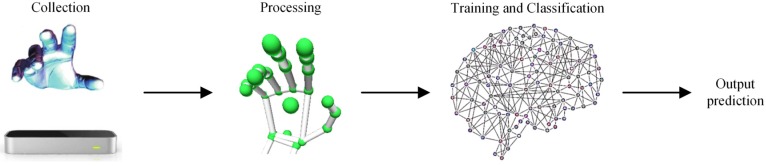
The architecture of the recognition system.

In this section, we present the architecture of our gesture recognition system. The systematic framework includes four parts, which are feature extraction, data collection and processing, two-layer BRNN, and model details. The architecture of gesture recognition system is shown in Figure 1, where the LMC is used to acquire the dynamic hand sequences, and data are processed by specific methods and sent into the two-layer BRNN model. Finally, the BRNN model outputs the recognition result.

### 2.1. Feature Extraction

The LMC can detect and track the human hand and wrist which appears 25 mm to 600 mm above the device plane, returning information on coordinate, velocity, acceleration, and others of five fingers, palm, and wrist. Each dynamic motion can be taken as a sequence of consecutive frames. Each frame can be considered as the combination of different hand features. In this part, we see a frame as a vector which consists of following features:

Fingertip distance

(1) $$D_{i}=\left\|\vec{OF_{i}}-\vec{OC}\right\|(i=1,2,3,4,5)$$

where, $D_{i}$ is the Euclidean distance between a fingertip coordinate and the palm coordinate, O is the origin of the three-dimensional coordinate system of Leap Motion, $F_{i}$ is the fingertip coordinate in the three-dimensional coordinate system of Leap Motion, C is the palm coordinate in the three-dimensional coordinate system of Leap Motion.

Fingertip angle

(2) $$\theta_{i}=∠\left(\vec{OF_{i}^{p}}-\vec{OC},\vec{OF_{i}}-\vec{OC}\right)(i=1,2,3,4,5)$$

where, $\theta_{i}$ is the angle between vector $\vec{CF_{i}^{p}}$ and vector $\vec{CF_{i}}$, $F_{i}{}^{p}$ is the projection of $F_{i}$ in the palm plane.

**Figure 2:**
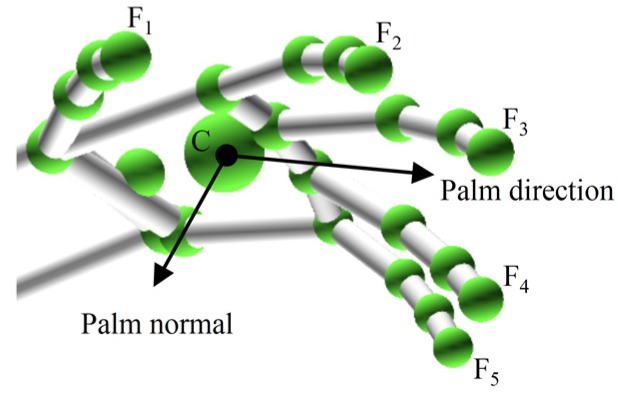
The hand model in the LMC.

Fingertip height

(3) $$H_{i}=sgn\left(\vec{F_{i}^{P}F_{i}}\cdot \vec{N}\right)*\left\|\vec{F_{i}^{p}F}\right\|(i=1,2,3,4,5)$$

where, $H_{i}$ is the vertical distance from point $F_{i}$ to palm plane, $\vec{N}$ is the palm normal (Figure 2), ⋅ is the dot product, ∗ is the multiplication sign.

The distance of adjacent fingertips

(4) $$DD_{i}=\left\|\vec{F_{i}F_{i+1}}\right\|(i=1,2,3,4)$$

The angle of adjacent fingertips

(5) $$\Theta_{i}=∠{\left(\vec{CF_{i}},\vec{CF_{i+1}}\right)}^{}(i=1,2,3,4)$$

where, $\Theta_{i}$ is the angle between vector $\vec{CF_{i}}$ and vector $\vec{CF_{i+1}}$.

The coordinate of the palm

(6) $$\left(x_{\mathrm{palm}},y_{\mathrm{palm}},z_{\mathrm{palm}}\right)$$

The above features are derived from the work [21,27,28] and they are extended and modified. Note that they are independent of each other and the final feature vector at time t is:

(7) $$X_{t}=\left\{D_{1},\cdots ,D_{5},\theta_{1},\cdots ,\theta_{5},H_{1},\cdots ,H_{5},DD_{1},\cdots ,DD_{4},\Theta_{1},\cdots ,\Theta_{4},x,y,z\right\}$$

This proposed vector not only solves the mislabeling problem caused by making dynamic hand gestures in different positions but also helps to recognize the difference which exists between adjacent fingers when people show different dynamic hand gestures.

A full dynamic gesture vector can be represented by:

(8) $$X=\left\{X_{1},X_{2},\cdots ,X_{j},\cdots ,X_{T}\right\}$$

where, T is the number of frames required to detect a full dynamic gesture and varies according to different dynamic gestures.

### 2.2. Data Collection and Processing

Dynamic gesture recognition relies on real-time and accurate gesture tracking. LMC uses binocular RGB high-definition cameras to improve gesture positioning accuracy and reduce the problems caused by occlusions between fingers. The infrared camera is used to filter images, which greatly reduces the impact of the background environment. Finally, a convolutional neural network is used to perform multi-layer convolution filtering on the image to extract feature data and provide it to the user. In terms of real-time and accuracy, LMC can provide gesture data more stably and accurately, providing a stable data guarantee for applications in some fields.

However, a challenge in dynamic hand gesture data collection is how to determine the start and end of a dynamic hand gesture. When LMC performs gesture acquisition, it obtains time-based dynamic sequences. Therefore, during the gesture collection, it is necessary to determine when the gesture execution starts and stops according to the threshold. This work uses the palm rotation threshold in a three-dimensional coordinate system and finger speed threshold to determine the start and end points of the dynamic hand gesture. The rotation of the palm needs to be obtained through comparison and calculation of the current frame and the historical frames, and the change in finger speed can be obtained through the library function that comes with LMC. The Algorithm 1 is shown below:
**Algorithm 1****If**(Hand is not empty) {  **If** (   sqrt (pow (frame. rotation Angle (last frame, x), 2)+      pow (frame. rotation Angle (last frame, y), 2)+      pow (frame. rotation Angle (last frame, z), 2))      >threshold1     **or**     fingertip. velocity > threshold2   )    Start collecting dynamic hand gestures  **Else**    Stop collecting dynamic hand gestures }**Else**  Stop collecting dynamic hand gestures

LMC allows access to a wide variety of features contained in the original dataset. After extraction and calculation, and obtaining specific features, the high-dimensional hand gesture sequences will make the model too computationally intensive in learning. In addition, every gesture in the acquisition process will have problems such as inconsistent shapes and inconsistent range sizes. Therefore, data need to be pre-processed, so that it is standardized to eliminate noise interference and reduce model learning costs. The same attribute of all samples is normalized separately, and all the samples become a series of data with a mean of 0 and a variance of 1.

### 2.3. Two-Layer Bidirectional Recurrent Neural Network

#### 2.3.1. The Basic Long Short-Term Memory Unit

**Figure 3:**
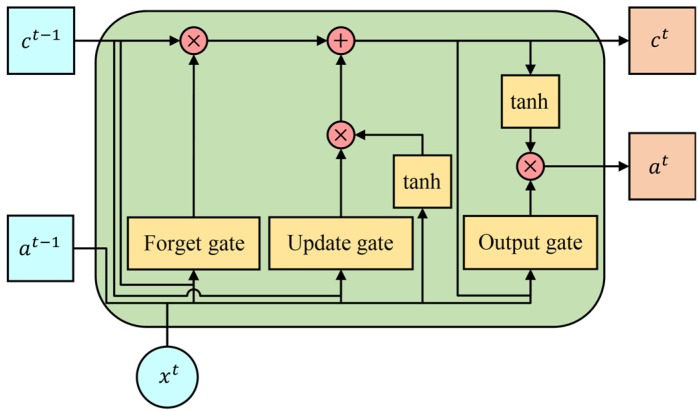
A basic Long Short-Term Memory unit with peephole.

The Long Short-Term Memory unit, as shown in Figure 3, is seen as the memory block and consists of three multiplicative gates which are input gate (update gate), output gate, and forget gate in the network. Additionally, the memory block also includes memory cells that are self-connected and save the temporal information of the network. The input gate is responsible for processing the input of the information. The output gate generates the flow of the output of cell activations to the rest of the units. The forget gate adaptively forgets some information in the memory cell. At time t, the formulas in the unit are defined as follow:

(9) $${\tilde{c}}^{t}=tanh\left(W_{c}\cdot \left[a^{t-1},x^{t}\right]+b_{c}\right)=tanh\left(W_{ca}\cdot a^{t-1}+W_{cx}\cdot x^{t}+b_{c}\right)$$

(10) $$\Gamma_{u}=\sigma \left(W_{u}\cdot \left[a^{t-1},x^{t},c^{t-1}\right]+b_{u}\right)=\sigma \left(W_{ua}\cdot a^{t-1}+W_{ux}\cdot x^{t}+W_{uc}\cdot c^{t-1}+b_{u}\right)$$

(11) $$\Gamma_{f}=\sigma \left(W_{f}\cdot \left[a^{t-1},x^{t},c^{t-1}\right]+b_{f}\right)=\sigma \left(W_{fa}\cdot a^{t-1}+W_{fx}\cdot x^{t}+W_{fc}\cdot c^{t-1}+b_{f}\right)$$

(12) $$c^{t}=\Gamma_{u}⊙{\tilde{c}}^{t}+\Gamma_{f}⊙c^{t-1}$$

(13) $$\Gamma_{o}=\sigma \left(W_{o}\cdot \left[a^{t-1},x^{t},c^{t}\right]+b_{o}\right)=\sigma \left(W_{oa}\cdot a^{t-1}+W_{ox}\cdot x^{t}+W_{oc}\cdot c^{t}+b_{o}\right)$$

(14) $$a^{t}=\Gamma_{o}⊙tanhc^{t}$$

where, $\Gamma_{u}$, $\Gamma_{o}$, $\Gamma_{f}$, and ${\tilde{c}}^{t}$ are the input gate, the output gate, the forget gate, and the new memory cell, respectively. The symbols $b_{u}$, $b_{f}$, $b_{o}$ and $b_{c}$ are bias vectors, respectively. Then $a^{t}$, $c^{t}$, $a^{t-1}$ and $c^{t-1}$ are the output of current block, final memory cell, output of previous block and previous memory cell. These vectors have the same length. $W_{u}$, $W_{o}$, $W_{f}$ and $W_{c}$ are the weights of the input gate, the output gate, the forget gate, the new memory cell, and the prediction, respectively. tanh is the hyperbolic tangent function. g is the softmax function. σ is the logistic sigmoid activation function. ⊙ is the element-wise product of the vectors.

#### 2.3.2. Bidirectional Recurrent Neural Network

**Figure 4:**
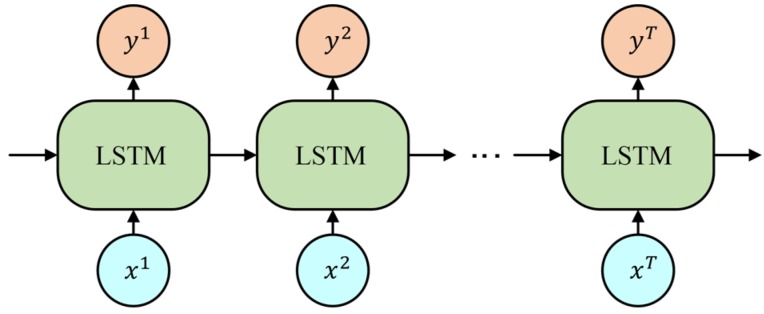
Example of connection between single-layer LSTM network.

The common Recurrent Neural Network provides an extremely useful method to handle time-based sequences which shows correlations between closely linked data elements in the sequence. Figure 4 shows a basic Recurrent Neural Network architecture with three units. Meanwhile, the previous Long Short-Term Memory unit provides its memory cell and output for the current unit as time goes on. In this figure, the input vector x which contains T frames is input into the network frame by frame. Compared with some other time-based models like Multilayer perceptron and time delay neural networks [29], this structure utilizes most of the available input information, from the start frame to current frame, to output vector y without the limitation of using a fixed-format input.

**Figure 5:**
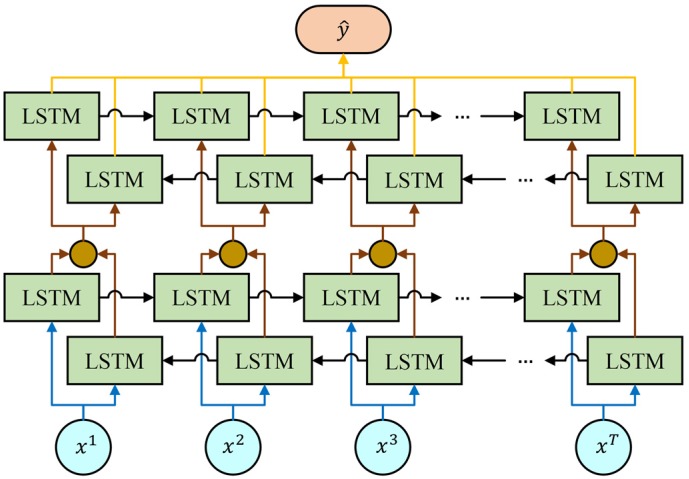
The two-layer Bidirectional Recurrent Neural Network. The outputs of the two corresponding LSTM units are added up in the first layer and sent to the second layer.

However, in the one-way Recurrent Neural Network, the current unit can only output the outcome based on the information of previous units. Especially, in some problems, the output of the current unit is not only related to previous units, but also related to the future units. In this case, it is possible to use two separate recurrent neural networks and then somehow merge outputs. In the single layer Bidirectional Recurrent Neural Network, one Recurrent Neural Network goes in a forward direction. On the contrary, another one goes in backward direction. At each time point, the input is provided to two independent Long Short-Term Memory units in opposite directions and they combine their outcomes based on the hidden state. In our work, we adopt this structure to build a two-layer BRNN and combine outcomes of the final Long Short-Term Memory unit of both networks (Figure 5). There are many other merging procedures to combine outcomes and it is generally not clear how to merge network outcomes in an optimal way since different networks trained on the same data can no longer be regarded as independent [29]. The formulas of the basic BRNN unit at time t are given by the following equations:

(15) $$\vec{{\tilde{c}}^{t}}=tanh\left({\vec{W}}_{ca}\cdot {\vec{a}}^{t-1}+{\vec{W}}_{cx}\cdot x^{t}+{\vec{b}}_{c}\right)$$

(16) $$\overset{\leftarrow }{{\tilde{c}}^{t}}=tanh\left({\overset{\leftarrow }{W}}_{ca}\cdot {\overset{\leftarrow }{a}}^{t-1}+{\overset{\leftarrow }{W}}_{cx}\cdot x^{t}+{\overset{\leftarrow }{b}}_{c}\right)$$

(17) $${\vec{\Gamma }}_{u}=\sigma \left({\vec{W}}_{ua}\cdot {\vec{a}}^{t-1}+{\vec{W}}_{ux}\cdot x^{t}+{\vec{W}}_{uc}\cdot {\vec{c}}^{t-1}+{\vec{b}}_{u}\right)$$

(18) $${\overset{\leftarrow }{\Gamma }}_{u}=\sigma \left({\overset{\leftarrow }{W}}_{ua}\cdot {\overset{\leftarrow }{a}}^{t-1}+{\overset{\leftarrow }{W}}_{ux}\cdot x^{t}+{\overset{\leftarrow }{W}}_{uc}\cdot {\overset{\leftarrow }{c}}^{t-1}+{\overset{\leftarrow }{b}}_{u}\right)$$

(19) $${\vec{\Gamma }}_{f}=\sigma \left({\vec{W}}_{fa}\cdot {\vec{a}}^{t-1}+{\vec{W}}_{fx}\cdot x^{t}+{\vec{W}}_{fc}\cdot {\vec{c}}^{t-1}+{\vec{b}}_{f}\right)$$

(20) $${\overset{\leftarrow }{\Gamma }}_{f}=\sigma \left({\overset{\leftarrow }{W}}_{fa}\cdot {\overset{\leftarrow }{a}}^{t-1}+{\overset{\leftarrow }{W}}_{fx}\cdot x^{t}+{\overset{\leftarrow }{W}}_{fc}\cdot {\overset{\leftarrow }{c}}^{t-1}+{\overset{\leftarrow }{b}}_{f}\right)$$

(21) $${\vec{c}}^{t}={\vec{\Gamma }}_{u}⊙{\vec{\tilde{c}}}^{t}+{\vec{\Gamma }}_{f}⊙{\vec{c}}^{t-1}$$

(22) $${\overset{\leftarrow }{c}}^{t}={\overset{\leftarrow }{\Gamma }}_{u}⊙{\overset{\leftarrow }{\tilde{c}}}^{t}+{\overset{\leftarrow }{\Gamma }}_{f}⊙{\overset{\leftarrow }{c}}^{t-1}$$

(23) $${\vec{\Gamma }}_{o}=\sigma \left({\vec{W}}_{oa}\cdot {\vec{a}}^{t-1}+{\vec{W}}_{ox}\cdot x^{t}+{\vec{W}}_{oc}\cdot {\vec{c}}^{t}+{\vec{b}}_{o}\right)$$

(24) $${\overset{\leftarrow }{\Gamma }}_{o}=\sigma \left({\overset{\leftarrow }{W}}_{oa}\cdot {\overset{\leftarrow }{a}}^{t-1}+{\overset{\leftarrow }{W}}_{ox}\cdot x^{t}+{\overset{\leftarrow }{W}}_{oc}\cdot {\overset{\leftarrow }{c}}^{t}+{\overset{\leftarrow }{b}}_{o}\right)$$

(25) $${\vec{a}}^{t}={\vec{\Gamma }}_{o}⊙{\vec{tanh\;c}}^{t}$$

(26) $${\overset{\leftarrow }{a}}^{t}={\overset{\leftarrow }{\Gamma }}_{o}⊙{\overset{\leftarrow }{tanh\;c}}^{t}$$

the output of the two-layer BRNN is defined as follows:

(27) $$\hat{y}=softmax({\vec{W}}_{y}\cdot \sum_{t=1}^{T}{\vec{a}}^{t}+{\overset{\leftarrow }{W}}_{y}\cdot \sum_{t=1}^{T}{\overset{\leftarrow }{a}}^{t}+b_{y})$$

where, → represents the forward Recurrent Neural Network and ← represents the backward Recurrent Neural Network. The scales of these four RNNs are the same.

### 2.4. Model Details

#### 2.4.1. Loss Function

The loss function is defined as below:

(28) $$L^{t}\left({\hat{y}}^{t},y^{t}\right)=-y^{t}\cdot log\left({\hat{y}}^{t}\right)$$

(29) $$L\left(\hat{y},y\right)=\frac{1}{m}\sum_{i=1}^{m}\sum_{t=1}^{T}L^{t}\left({\hat{y}}^{t},y^{t}\right)$$

where, $\hat{y}$ is prediction of the sample X, y is the label of the sample X, and $y^{t}$ is the label of the $X_{t}$ in the sample X. In addition, m is the number of samples and T is the length of one sample based on the time sequence. Further, ⋅ is the inner product. This formulation is obtained by referring to and modifying the cross-entropy proposed in [30].

#### 2.4.2. Learning Rate

Learning rate is the most important hyper-parameter in all kinds of neural networks. Consequently, Cyclical Learning Rate method was adopted in this work, which can eliminate the need to vary the learning rate yet achieve near optimal classification accuracy. Instead of monotonically decreasing the learning rate, CLR lets the learning rate vary cyclically between reasonable boundary values which are achieved through, linearly, increasing the learning rate for a few epochs. The corresponding codes are the given as:

(30) $$s=\frac{Iterations}{Batchsize}$$

(31) $$c=\left[1+\frac{en}{2\cdot s}\right]$$

(32) $$p=\left|\frac{en}{s}-2\cdot c+1\right|$$

(33) $$LR=LR_{min}+\left(LR_{max}-LR_{min}\right)\cdot max\left(0,1-p\right)$$

where [*x*] is to take the largest integer less than *x*, |*x*| is to take the absolute value of *x*, and max(*x*, *y*) is to take the larger of *x* and *y*. The *en* is the sequence number of epochs. More details can be found in [31].

## 3. Experimental Results and Discussion

This section shows a series of dynamic hand gesture recognition experiments. The systematic framework includes six parts: dynamic gesture datasets, selection of the optimal dropout rate on two datasets, experiment results on ASL dataset, experiment results on Handicraft-Gesture dataset, k-fold and Leave-One-Out cross-validation, and comparisons between our results with the works found in the literature.

### 3.1. Dynamic Gesture Datasets

**Figure 6:**
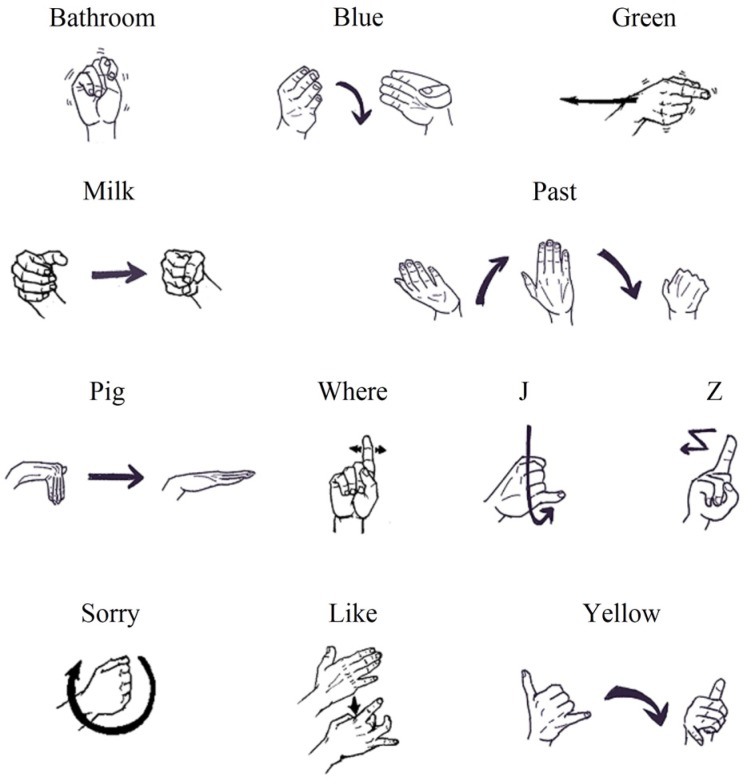
The illustration of twelve gestures of the ASL dataset.

**Figure 7:**
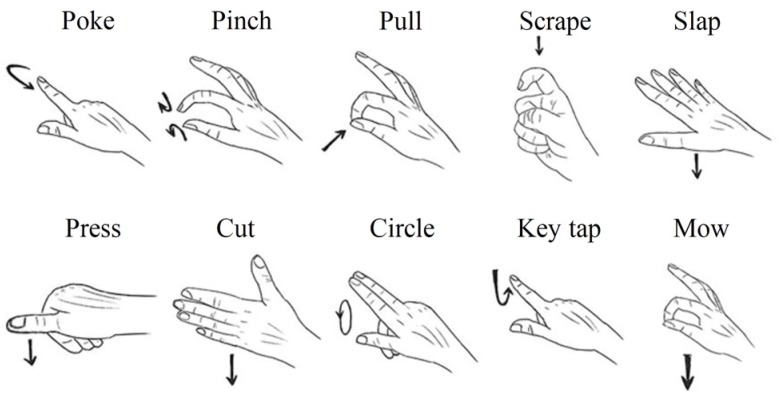
The illustration of ten gestures of the Handicraft-Gesture dataset.

Here, we created two new datasets composed of 12 hand gestures and 10 hand gestures, respectively. They are called ASL Dataset (Figure 6) and Handicraft-Gesture Dataset [21] (Figure 7). In particular, the ASL Dataset is picked from the American Sign Language and the other dataset is chosen from the paper [21].

#### 3.1.1. ASL Dataset

Currently, most of datasets are based on images. In order to test the performance of the model we proposed, we built this dataset with an LMC. This dataset contains a series of gestures from ASL. The 12 gestures are bathroom, blue, green, j, like, milk, past, pig, sorry, where, yellow, and z. The arrows are the motion trajectories of the hand or the fingers.

This work is mainly focused on single-hand dynamic gestures. In order to compare with other works, like [21,23] which has the dataset containing bathroom, blue, finish, green, hungry, milk, past, pig, store, where, j, and z, we replaced finish, hungry, and store with sorry, like, and yellow which are nearly equivalent to them in terms of difficulty.

#### 3.1.2. Handicraft-Gesture Dataset

In order to evaluate the proposed model with more different dynamic hand gestures, we introduced the Handicraft-Gesture dataset. This dataset includes 10 gestures, namely poke, scrape, circle, pull, pinch, slap, cut, key type, press, and mow.

All gestures are dynamic and based on a single hand, coming from four different people aged 25. In order to compare with other literatures, there are two types of ASL datasets which contain 360 and 480 samples, respectively. In particular, the dataset with 480 samples is from [23] which has a dataset composed of 720 static hand sequences and 480 dynamic hand sequences. In the one that contains 360 samples, each type of gesture has 30 samples that are performed by four different persons. According to [21], 70% of the data are used as the training set and 30% are used as the testing set. In the other one, each type of gesture has 40 samples that are performed by four different persons. According to [23], 65% of the data are used as the training set and 35% are used as the testing set. The Handicraft-Gesture dataset is composed of 300 samples and each type of gesture has 30 samples that are performed by four different persons. According to [21], 70% of the data are used as the training set and 30% are used as the testing set.

### 3.2. Selection of the Optimal Dropout Rate on Two Dataset

Although BRNN has shown excellent ability to capture deep information between sequences, BRNN architectures are prone to overfit [32,33]. In order to avoid the problem of overfitting, one of the most useful techniques in neural networks, namely dropout regularization, was taken. Several experiments on the selection of the dropout rate were conducted.

**Figure 8:**
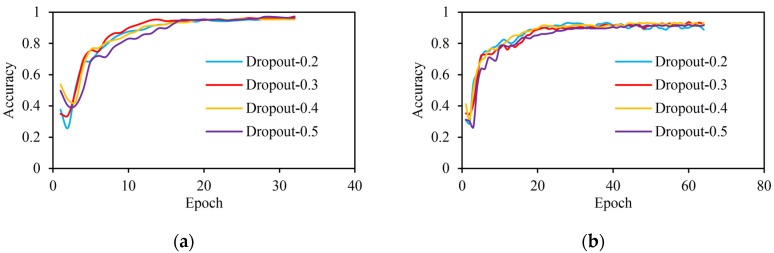
Accuracies on datasets by varying the dropout rate. (**a**) Accuracies on the ASL dataset; (**b**) Accuracies on the Handicraft-Gesture dataset.

The Figure 8a shows that the best dropout rate for the ASL dataset is between 0.3 and 0.4, and the Figure 8b indicates that the best dropout rate for the Handicraft-Gesture dataset is between 0.4 and 0.5. However, as the number of iterations increases, the dropout rate still needs to be tuned properly in these two ranges.

### 3.3. Experiment Results on ASL Dataset

Using an LMC and a computer with an Intel i5 3.2GHz CPU and 8GB RAM, experiments were performed to estimate the performance of the proposed two-layer BRNN model. The model was trained using cross-entropy loss function, Adam gradient descent algorithm [34], and varying learning rate. The model was implemented by using a framework in C++, written by ourselves. 

**Figure 9:**
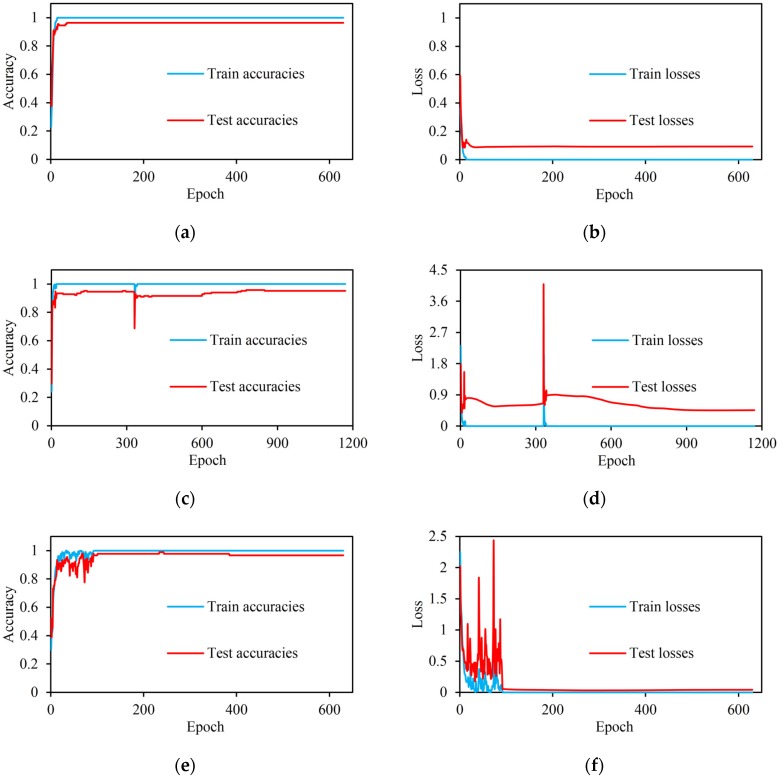
Curves for the accuracies and losses of the training and testing sets. (**a**) The accuracies of the ASL dataset with 360 samples; (**b**) The losses of the ASL dataset with 360 samples; (**c**) The accuracies of the ASL dataset with 480 samples; (**d**) The losses of the ASL dataset with 480 samples; (**e**) The accuracies of the Handicraft-Gesture dataset with 300 samples; (**f**) The losses of Handicraft-Gesture dataset with 300 samples.

Figure 9a–f shows changes in the recognition accuracy of the model on the training and testing sets as the number of iterations increases and loss curves which represent the sum of the errors provided for each training and test sample. On the ASL dataset with 360 samples, after 630 epochs (18, 900 iterations), the curve of the train accuracy converges to 100% and the curve of the test accuracy converges to 96.2963%, respectively. On the ASL dataset with 480 samples, after 1170 epochs (70, 200 iterations), the curve of the train accuracy converges to 100% and the curve of the test accuracy converges to 95.238%, respectively. In Figure 9c,d, a sudden and unexpected leap of value happens over the epoch of 340 for accuracies and losses on both training and testing sets. To explore the cause we did another three types of experiments, each performed five times. The first experiment used the CLR but did not use the dropout, the second experiment used the dropout but did not use the CLR, and the third experiment did not use any of them. The result shows that the leap happens randomly when the model uses the dropout and CLR. It seems to be a random and transient instability because this leap only happens when the learning rate varies during the first period (0–400 epoch), and after this the model will consistently converge with smaller learning rate. This leap happens infrequently, and it does not appear in experiments on the other two datasets.

In order to better evaluate the proposed approach, three significant metrics were taken, namely Precision, Recall, and F1-score. As a standard, these indexes can be used to measure the performance of the model [35]. The results are presented in Table 1.

**Figure 10:**
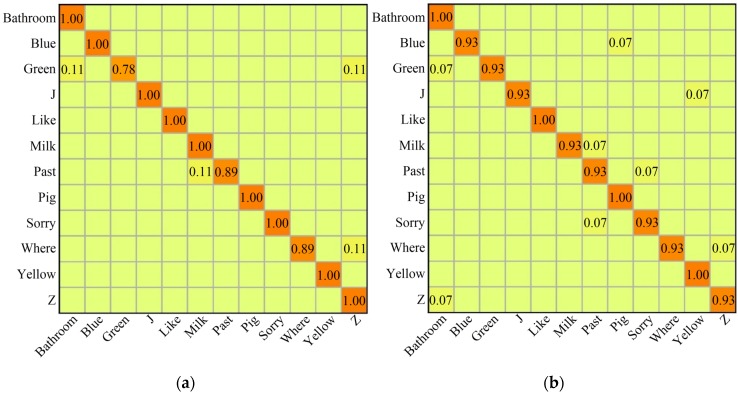
Heat-maps of the confusion matrix for the recognition of the ASL dataset. (**a**) The heat-map of the ASL dataset with 360 samples; (**b**) The heat-map of the ASL dataset with 480 samples.

In addition, heat-maps of the confusion matrix were also computed and drawn (Figure 10). Each column of the confusion matrix represents the predictive value while each row represents the real value. In the confusion matrix, each diagonal element represents the prediction accuracy of the corresponding row identifier. The elements below or above the diagonal are misclassified. As depicted in Figure 10, for each gesture, there is a low accuracy for gesture ‘green’ (78%) on the dataset with 360 samples. However, the accuracy of each gesture is above 93% on the dataset with 480 samples, which is a good performance.

### 3.4. Experiment Results on Handicraft-Gesture Dataset

**Figure 11:**
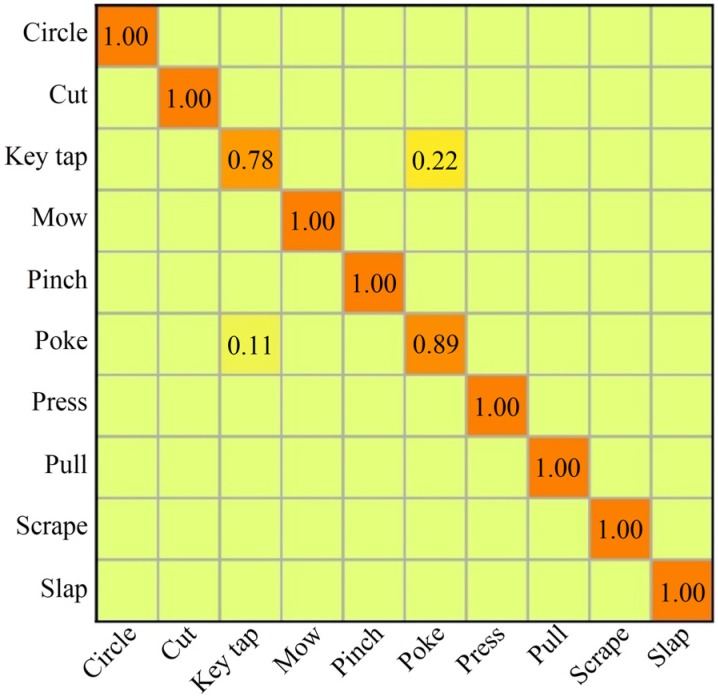
The heat-map of the confusion matrix for the recognition of the Handicraft-Gesture dataset.

Similar experiments and evaluations were executed on the Handicraft-Gesture dataset. As shown in Figure 9, after 630 epochs (15,750 iterations), the curve of the train accuracy converges to about 100% and the curve of the test accuracy converges to about 96.6667%, respectively. In addition, Table 2 shows the F1-score of 96.6563% and Figure 11 describes the heat-map of the confusion matrix for recognizing hand gestures on this dataset.

Except for low accuracies for gesture ‘key tap’ (78%) and ‘poke’ (89%), all other accuracies are 100%. It is clear that, at some moments, key tap and poke have similar hand shapes and the same motion trajectories in different directions. Different people make this gesture with different force and directions. For this reason, the key tap and poke are misclassified in some cases.

### 3.5. K-Fold and Leave-One-Out Cross-Validation

As a statistical analysis method, cross-validation is commonly used to verify the performance of the recognition model. In the work, we adopted the 5- fold cross-validation, 10-fold cross-validation, and Leave-One-Out cross-validation to verify the model. In the 5-fold cross-validation, the dataset is divided into 5 subsets. In each subset, there are 72 samples for the ASL dataset with 360 samples, 96 samples for the ASL dataset with 480 samples, and 60 samples for the Handicraft-Gesture dataset. In 10-fold cross-validation, the dataset is divided into 10 subsets. In each subset, there are 36 samples for the ASL dataset with 360 samples, 48 samples for the ASL dataset with 480 samples, and 30 samples for the Handicraft-Gesture dataset. In the Leave-One-Out cross-validation, for each dataset, one sample is used as the testing set and the others are used as the training set. Leave-One-Out cross-validation is robust, but it is computationally expensive. In order to reduce the computation time, we chose to use pre-training (Transfer Learning) [36] to conduct Leave-One-Out cross-validation. In our experiments, for each dataset, pre-training starts with a model resulted from about 200 epochs of computation on the same dataset. We noticed that the model converges quickly on these datasets. Therefore, we only ran nearly five epochs (nearly 150 iterations) for the Leave-One-Out cross-validation experiments to obtain comparably good results. The results are shown in Table 3, Table 4 and Table 5.

### 3.6. Comparisons

Firstly, we compared the proposed method with several significant works [21,23,37,38] on the ASL dataset. American Sign Language is one of the most important communication tools between the deaf–mute and contains many challenging dynamic hand gestures. Most of works chose a subset of the American Sign Language to build dataset and tested recognition systems. 

Table 6 shows that the proposed method surpasses the accuracies of three works on the ASL dataset and, compared to [23], we obtained similar results on the dataset. In particular, the overall accuracy in [23] is 96.4102%, but the dataset contains 18 static hand gestures (720 samples) and 12 dynamic hand gestures (480 samples). We removed the static hand gesture, and the accuracy of the dynamic hand gesture is calculated to be 95.2%. The recognition of static hand gestures has matured in recent years and, sometimes, the accuracy is up to 100%. Therefore, it is reasonable for us to compare with it. The other works are all based on the same or a smaller sample size, and they have different models and collection approaches of dynamic hand gestures.

Furthermore, this work also compared the proposed method with the work in [21] on Handicraft-Gesture dataset and, as shown in Table 7, our method achieved better performance. In addition, in its confusion matrix, it has low accuracies in the recognition of gesture ‘pinch’ (82.5%) and ‘pull’ (87.5%). However, our work only gets a low accuracy in the recognition of ‘key tap’ (78%) (Figure 11).

The computation time of our experiments is shown in the Table 8.

## 4. Conclusions

In this work, a recognition system of dynamic hand gesture recognition method is presented. It is based on a two-layer Bidirectional Recurrent Neural Network. In addition, an efficient dynamic gesture acquisition framework is proposed, and 26 discriminative features based on angles, positions, and distances between fingers are used. The system was able to obtain high accuracy results and our method demonstrated similar or better performance than several works on ASL and Handicraft-Gesture datasets. Future work will be focused on larger and more difficult dynamic hand gestures, combining two or more LMCs to capture dynamic hand gesture sequences. Furthermore, we are also working on creating a new dataset that includes more challenging dynamic hand gestures from ASL and other sign languages which supports multiple formats of data, such as time-based sequences and depth images. This dataset will serve as benchmark for testing on different models.

## Figures and Tables

**Table 1:** The performance of the proposed approach on the ASL dataset.

Type	Accuracy	Precision	Recall	F1-Score
360 samples	96.2963%	96.8182%	96.2963%	96.2674%
480 samples	95.238%	95.546%	95.238%	95.274%

**Table 2:** The performance of the proposed approach on the Handicraft-Gesture dataset.

Accuracy	Precision	Recall	F1-Score
96.6667%	96.75%	96.6667%	96.6563%

**Table 3:** Recognition accuracies (%) for the three datasets by using the 5-fold cross-validation method.

	ASL Dataset (360 Samples) (%)	ASL Dataset (480 Samples) (%)	Handicraft-Gesture Dataset (%)
1	95.83	90.63	93.33
2	95.83	94.79	83.33
3	84.72	90.63	90
4	94.44	94.79	83.33
5	95.83	97.93	93.33
Average:	93.33	93.75	88.66

**Table 4:** Recognition accuracies (%) for the three datasets by using the 10-fold cross-validation method.

	ASL Dataset (360 Samples) (%)	ASL Dataset (480 Samples) (%)	Handicraft-Gesture Dataset (%)
1	94.44	97.92	100
2	88.89	93.75	93.33
3	91.67	95.83	96.67
4	91.67	89.53	86.67
5	97.2	91.67	86.67
6	97.2	87.5	83.33
7	86.1	91.67	76.67
8	100	97.92	96.67
9	97.2	97.92	86.67
10	97.2	91.67	93.33
Average:	94.1	93.5	90

**Table 5:** Recognition accuracies (%) for the three datasets by using the Leave-One-Out cross-validation method.

	ASL Dataset (360 Samples) (%)	ASL Dataset (480 Samples) (%)	Handicraft-Gesture Dataset (%)
Average:	98.33	98.13	92

**Table 6:** Comparison of the accuracy among significant works on the ASL dataset.

Method	Dataset	Accuracy
This paper’s method	ASL (360 samples)	96.3%
This paper’s method	ASL (480 samples)	95.2%
Deep Recurrent Neural Network on Depth Features [23]	ASL (480 samples)	95.2%
Hidden Conditional Neural Field on Depth Features [21]	ASL (360 samples)	89.5%
Action Graph on Silhouette Features [37]	ASL (360 samples)	87.7%
SVM on Random Occupancy Pattern Features [38]	ASL (334 samples)	88.5%

**Table 7:** Comparison of the accuracy among significant works on the Handicraft-Gesture dataset.

Method	Dataset	Accuracy
This paper’s method	Handicraft-Gesture (300 samples)	96.7%
Hidden Conditional Neural Field on Depth Features [21]	Handicraft-Gesture (300 samples)	95%

**Table 8:** The computation time of the experiments.

Dataset	Item	Epoch	Time(h)
ASL dataset with 360 samples	single experiment (7:3)	620	2
	5-fold cross-validation (4:1)	612	9.7
	10-fold cross-validation (9:1)	612	29.52
	Leave-One-Out cross-validation (359:1)	5	5.4
ASL dataset with 480 samples	single experiment (13:7)	1170	4.4
	5-fold cross-validation (4;1)	720	13.1
	10-fold cross-validation (9:1)	810	31.3
	Leave-One-Out cross-validation (479:1)	5	9.5
Handicraft-Gesture dataset with 300 samples	single experiment (7:3)	630	1.8
	5-fold cross-validation (4:1)	630	8.8
	10-fold cross-validation (9:1)	810	24.9
	Leave-One-Out cross-validation (299:1)	5	3.8
